# Sentinel Lymph Node Biopsy Versus Elective Neck Dissection in Carcinoma of the Tongue and Floor of the Mouth

**DOI:** 10.3390/cancers17193098

**Published:** 2025-09-23

**Authors:** Carolin Naegeli-Pullankavumkal, Tamara Manser, Tarun Mehra, Niels Jan Rupp, Thomas Gander, Martin W. Huellner, Martin Lanzer

**Affiliations:** 1 Department of Cranio-Maxillo-Facial and Oral Surgery, University Hospital Zürich, 8091 Zürich, Switzerland; 2 Comprehensive Cancer Center Zürich, University Hospital Zürich, 8091 Zürich, Switzerland; 3 Department of Pathology and Molecular Pathology, University Hospital Zürich, 8091 Zürich, Switzerland; 4 Department of Nuclear Medicine, University Hospital Zürich, 8091 Zürich, Switzerland

**Keywords:** sentinel lymph node biopsy, elective neck dissection, carcinoma of the tongue, carcinoma of floor of the mouth, survival, recurrence rates

## Abstract

**Background/Objectives:** Lymph node metastases represent the most important prognostic factor for survival in patients with localized squamous cell carcinoma (SCC) of the oral cavity. In patients with carcinoma of the tongue or floor of the mouth, elective neck dissection (END) has been the standard of care, but it has been replaced at many centers by sentinel lymph node biopsy (SLNB). This study’s purpose was to measure and compare recurrence rates between END and SLNB in patients with early SCC of the oral cavity. **Methods:** Patients were included in this retrospective monocenter cohort study, performed at the University Hospital of Zürich, if they underwent either END or SLNB for oral squamous cell carcinoma (OSCC) of the tongue or floor of the mouth between January 2008 and December 2018. Only patients with early-stage tumors and a clinically negative neck (T1 or T2; cN0) who had a follow-up period of at least 5 years were included. Patients with a T3 or T4 tumor or a clinically positive neck were excluded, as were those who previously underwent therapy for another head and neck carcinoma. The predictor variable was the surgical treatment used, with subjects divided into two groups: END and SLNB. The main outcome variable was the time to recurrent disease, defined as the time between the primary surgical treatment and the diagnosis of recurrence. The covariates were primary tumor location, pT status, pN status, histopathological grade and postoperative radiotherapy. We used descriptive analysis, univariate analysis and the logrank test, with a *p*-value < 0.05 considered statistically significant. We deliberately refrained from multivariate analysis due to insignificant statistical results in the univariate analysis. **Results:** In total, 82 patients (46 male, 36 female; median age at the time of surgery: 58.9 years) were included, with a median observation period of 4.3 years. The main primary tumor location was the floor of the mouth (62.2%). The SLNB cohort had smaller primary tumors in comparison to the END cohort (20% vs. 37% pT2, respectively). Furthermore, pN+ disease was more prevalent in the END group in comparison to the SLNB group (81.8% vs. 74.1% pN0, respectively). Recurrence-free survival was not significantly different between the two groups. Subgroup analysis demonstrated a higher risk of recurrent disease in pN+ groups undergoing SLNB compared to those undergoing END. **Conclusions:** We demonstrate that SLNB is a useful tool for assessing lymph nodes of the neck. In cases of pN+ necks after SLNB and subsequent neck dissection, patients need to be closely followed up with due to the risk of recurrent disease.

## Simple Summary

This study examined two different surgical strategies for patients with early-stage squamous cell carcinoma of the oral cavity, focusing on cancers of the tongue and floor of the mouth. Traditionally, elective neck dissection (END) has been the standard treatment to remove potential lymph node metastases. In recent years, many centers have introduced sentinel lymph node biopsy (SLNB) as a less invasive alternative. To compare these approaches, the medical records of patients treated between 2008 and 2018 were analyzed. The results showed that SLNB achieved survival outcomes comparable to END in patients with small tumors (T1–T2) and no clinical signs of lymph node involvement (cN0). Nevertheless, the analysis indicated that patients with hidden lymph node metastases (pN+) who received delayed neck treatment after SLNB may face a higher risk of cancer recurrence. Further clinical studies are needed to clarify this potential risk and to determine the best treatment strategy for these patients.

## 1. Introduction

Squamous cell carcinoma (SCC) of the head and neck is the seventh most common cancer worldwide, and its incidence is rising [1]. The neck status in SCC of the oral cavity is the most critical prognostic factor for patient survival [2]. Therefore, correct staging of the neck is of utmost importance. The reported presence of occult cervical lymph node metastases, defined as pathologically identifiable metastases that are undetectable upon clinical examination or imaging, in patients with oral SCC (OSCC) varies between 14% and 42% [3,4,5,6,7,8].

Except in the case of T1–2 glottic tumors, elective neck dissection (END) remains the standard of care for the diagnostic nodal assessment of the ipsilateral neck. However, this approach can lead to substantial overtreatment and unnecessary morbidity. Consequently, many national guidelines, including those of the United States (National Comprehensive Cancer Network, NCCN), have adopted sentinel lymph node biopsy (SLNB) as the preferred method for nodal assessment of the neck [9].

Lymph node metastases are most often found in the sentinel lymph node (SLN), which is defined as the first draining lymph node of the primary tumor. The SLN is located using scintigraphy, wherein a radionuclide is injected into the peritumoral tissue. Occasionally, more than one SLN is found [10]. Through the SLNB method, occult lymph node metastases can be detected and histopathologically verified. However, it is important to note that SLNB is a diagnostic procedure, not a therapeutic one.

Occult metastases in the SLN are classified into three categories based on their size: macrometastases are defined as metastases of at least 2 mm, micrometastases as smaller than 2 mm and isolated tumor cells (ITCs) as smaller than 0.2 mm. ITCs can be further divided into single tumor cells and small cell clusters. Unlike micrometastases, ITCs typically do not show signs of proliferation, nor extravasation and stromal reaction [11].

SLNB was developed for patients with breast cancer and malignant melanoma [12], though it has since been applied to oral cavity cancer. In breast cancer and malignant melanoma, SLNB is also used to determine the necessity of further treatment [13,14].

The SLN is identified through a sensitive procedure that requires the cooperation of experienced surgeons, nuclear medicine physicians and pathologists [15]. However, there are instances where no lymph nodes are detected, which can occur due to an incorrect injection technique, a blockage or a drainage path of an unexpected type [10]. It is important to mention that SLNB, as a less invasive procedure, is non-inferior in terms of diagnostic accuracy compared to the standard of care, END.

At the University Hospital of Zürich, SLNB for stage 1 or 2 cancers of the floor of the mouth or tongue was introduced in 2014, before which patients were usually treated with END. After 2015, SLNB became predominant.

The investigators hypothesize that recurrence-free survival of patients diagnosed using SLNB is non-inferior to that of patients diagnosed using END. The purpose of our study was to measure and compare recurrence rates between different strategies, END and SLNB, in patients with early SCC of the tongue and floor of the mouth.

The specific aims of this study are to verify the diagnostic capability of SLNB and identify risk factors of recurrent disease.

## 2. Patients and Methods

### 2.1. Study Design/Sample

This cohort study was conducted as a retrospective monocenter study. Patient data were included if the individuals had undergone either END or SLNB for SCC of the floor of the mouth or tongue at the University Hospital of Zürich between January 2008 and December 2018. Only patients with early-stage tumors (T1 or T2, cN0 and cM0, based on the 7th edition of the TNM staging system) were included. Patients with metastatic, N+, or locally advanced disease (cT3/cT4) were excluded, as were those who had previously undergone therapy for another head and neck carcinoma. Patients were included if they had an uneventful follow-up period longer than 5 years; in case of recurrent disease, all patients were included. The primary endpoint was recurrence-free survival (RFS), defined as the time from the date of primary surgery to the first documented local, regional, or distant recurrence, or death from any cause, whichever occurred first. Patients without an event were censored at the date of last clinical follow-up or, if applicable, at the date of death from causes unrelated to the carcinoma without evidence of recurrence.

### 2.2. Variables

The primary predictor variable in our study is the type of surgical treatment. Patients were categorized into 2 groups: those who underwent END and those who underwent SLNB.

The primary outcome variable in our study is the time to recurrence, defined as the time between the first operation to combat the initial tumor, alongside initial END or SLNB, and the date of diagnosis of recurrence. Covariates include the date of initial diagnosis, date of primary treatment, primary tumor location, TNM classification, pathology results for the SLNB or END specimen, date, localization, extent of recurrence, and date of death.

### 2.3. Surgical Management

All patients were treated surgically at the Department of Oral and Maxillofacial Surgery, University Hospital Zürich. Each underwent primary tumor resection along with either SLNB or END. Pathological examination of the SLN and the primary tumor tissue included histological analysis of the whole lymph node specimen, including pancytokeratin (AE1/AE3) immunohistochemistry on additional blank sections. All patients had provided written informed consent for the retrospective use of their data for research purposes. The treatment plan for each patient had been discussed in turn by a multidisciplinary tumor board. Treatment recommendations were aligned with the latest national and international guidelines. Criteria for recommending adjuvant therapy, such as radiotherapy, included the presence of more than one positive lymph node and specific histopathological factors, such as perineural invasion and capsule penetration.

In patients who underwent END, the lymph nodes of the supraomohyoidal compartments, alongside level IV of the neck for those with tongue cancer, were removed.

SLNB was conducted as follows: Initially, dynamic lymphoscintigraphy was performed preoperatively to map the lymphatic drainage. This was followed by SPECT/CT (Single-Photon Emission Computed Tomography/Computed Tomography) to precisely locate the SLN. During surgery, a gamma probe was used to identify the SLNs. In addition, indocyanine green (ICG) was employed, particularly in floor of the mouth tumors, to thoroughly assess lymph nodes at level 1. The histological processing of the SLNs adhered to established protocols [16]. Upon identification of a positive SLN, the tumor tissue was categorized into macrometastases, micrometastases, or ITCs [11]. If either micrometastases or macrometastases were detected during the pathological processing of the SLN, END was conducted. The presence of ITCs entailed a thorough review and discussion of the patient’s situation among the interdisciplinary tumor board members to determine the appropriate further treatment of the neck.

After surgery, patients attended follow-up appointments in regular intervals for at least 5 years. During these appointments, any recurrence or metastatic disease was assessed and managed appropriately.

### 2.4. Covariates

The measured covariates are primary tumor location, pT status, pN status, histopathological grade and postoperative radiotherapy. The location of the tumor was determined based on the body parts (e.g., tongue) and side (left, right or center) involved. T and N status as well as grade was recorded based on the pathology reports and the current TNM system. Whether a patient received postoperative radiotherapy was discussed by the tumor board.

### 2.5. Data Collection Methods

To gather the data, all patients who underwent surgery for SCC of the floor of the mouth or tongue at the University Hospital Zürich between 2008 and 2018 were screened using the above-specified inclusion and exclusion criteria. Electronic health records of eligible patients were reviewed to extract demographic information and operative details, which were then stored in a secure, HIPAA-compliant database provided by the University Hospital. Data were deidentified to ensure patient privacy. The variables used in our study were obtained from surgical, pathology and diagnostic reports.

### 2.6. Data Analyses

The study sample was characterized using descriptive statistics. Time-based recurrence-free survival analysis was conducted using the Kaplan–Meier method, with statistical significance tested using the logrank test. Statistical significance was defined at a 2-sided alpha error of *p* < 0.05. Initially, overall survival analysis was planned, but it was later discontinued due to the limited number of recorded events (6 in END group and 3 in SLNB group).

Data were collected in Microsoft Excel Version 16.43 (Microsoft Corporation, Redmond, Washington, DC, USA). Statistical analysis was performed using SPSS Statistics version 26 (IBM Corporation, Armonk, NY, USA).

This study was approved by the cantonal ethics committee (Nr. 2018-02297 on 4 February 2019).

## 3. Results

In total, 82 patients were included in our study (55 in the SLNB group and 27 in the END group). The median duration of the observation period was 4.3 years overall (0.01–15.7 years), or 4.1 years (0.2–13.2 years) in the SLNB group and 4.8 years (0.01–15.7 years) in the END group. A detailed summary of the cohort can be found in Table 1. Among those included, 60.5% were male, with the median age being 58.9 years (51.4–71.5 years) and similar between groups. The two primary tumor locations were the tongue (62.2%) and floor of the mouth (37.8%). In the END group, the main cancer type was carcinoma of the floor of the mouth (66.7%), whereas it was carcinoma of the tongue in the SLNB group (76.4%). In approximately one quarter of the cases, the tumor extended to the midline, with a slightly higher proportion of tumors reaching the midline in the END group (33.3%) compared to the SLNB group (22.6%). In both groups, the primary tumor was mostly unilateral (SLNB group: 45.5% left, 47.3% right, 7.3% center; END group: 44.4% left, 51.9% right, 3.7% center).

The SLNB cohort had smaller primary tumors in comparison to the END cohort (20% vs. 37% pT2, respectively). Additionally, there was a higher incidence of pN+ disease in the END group compared to the SLNB group (81.8% vs. 74.1% pN0, respectively). Moreover, a higher proportion of less-differentiated tumors was observed in the END group than in the SLNB group (3.7% vs. 30.9% G1, 63.0% vs. 54.4% G2 and 33.3% vs. 14.5% G3, respectively). In our cohort, only one patient had ITCs and was treated with END after undergoing prior SLNB treatment.

Overall, 15 events of recurrence or metastasis were recorded in the patient cohort, with 13 in the SLNB group and 2 in the END group (Table 2). Of these recurrence events, 4 occurred at a nodal site and 11 at a local site.

Table 3 summarizes recurrence patterns. Local recurrence predominated, particularly in the SLNB cohort, whereas nodal failures were comparatively infrequent and no distant metastases occurred in the END group. One distant recurrence in the SLNB cohort appeared in the soft tissue of level VI/VII.

Among patients with recurrence or secondary metastatic disease, the median time to occurrence was lower in the SLNB group (3.9 years) compared to the END group (6.2 years).

**Figure 1:**
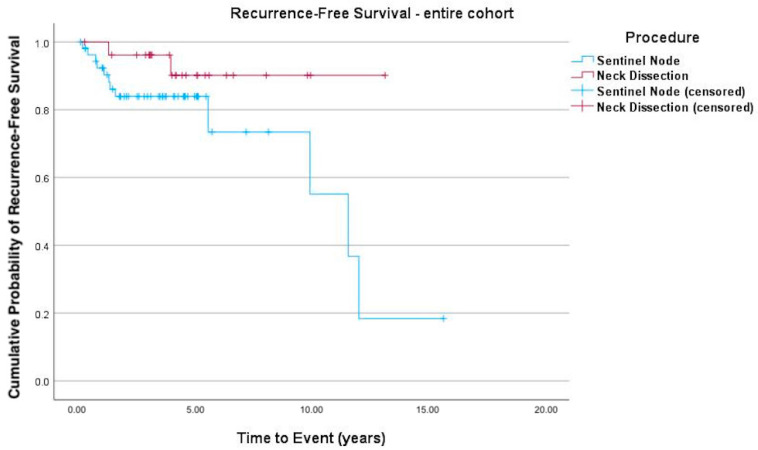
Kaplan–Meier curve depicting recurrence-free survival. There was no significant difference in recurrence-free survival in the patient cohort (logrank test *p* = 0.090), although there was a trend toward a higher recurrence rate with SLNB (Hazard Ratio (HR) = 3.4 (95% confidence interval (CI) 0.76–15.22; *p* = 0.11)).

**Figure 2:**
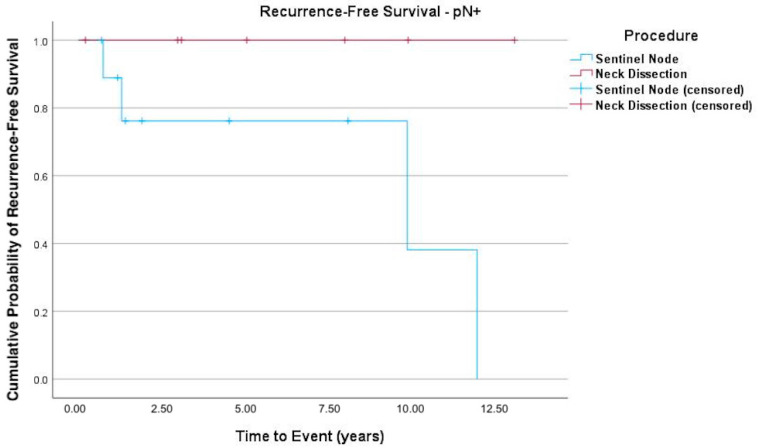
Kaplan–Meier curve of recurrence-free survival of pN+ patients. Subgroup analysis of patients diagnosed as pN+ after END or SLNB, with the latter exhibiting a higher likelihood of recurrent disease, albeit without significance (logrank test *p* = 0.053; HR = 58.2 (95% CI 0.018–189 388.43; *p* = 0.325)).

**Figure 3:**
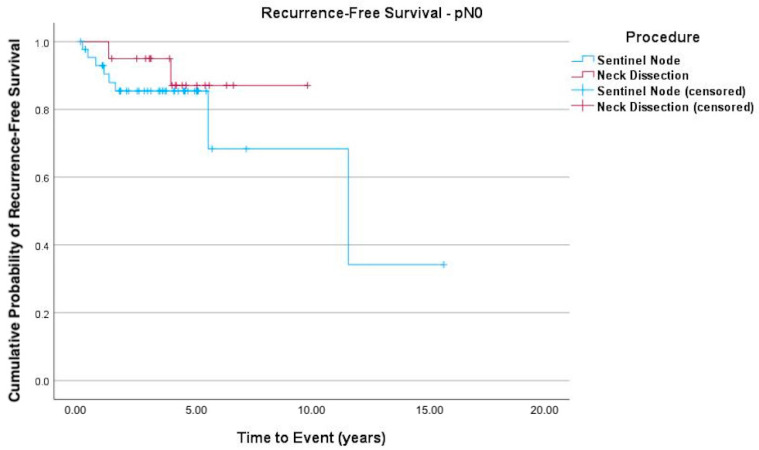
Kaplan–Meier curve of recurrence-free survival of pN0 patients. Patients with a pN0 neck after END or SLNB demonstrated no statistical difference in recurrent disease (logrank test *p* = 0.398; HR = 1.9 (95% CI 0.40–9.42; *p* = 0.406)).

The Kaplan–Meier curves (Figure 1, Figure 2 and Figure 3) indicate recurrence-free survival. In Figure 1, the recurrence-free survival of both groups (SLNB versus END) is compared. Figure 2 shows the survival of pN+ patients, while Figure 3 depicts that of patients with negative lymph nodes. Among the 55 SLNB patients, 42 (76.36%) experienced no recurrence or death events, with this also being the case for 25 of the 27 patients (92.6%) in the END group (logrank test *p* = 0.090).

In the subgroup analysis (Figure 2) of patients diagnosed as pN+ after END or SLNB, those in the latter group demonstrated a borderline higher likelihood of recurrent disease (logrank test *p* = 0.053).

## 4. Discussion

At the University Hospital Zürich, SLNB has been used since 2014 as a standard diagnostic tool for the neck in early SCC of the tongue or floor of the mouth (T1 or T2) and for clinically negative necks (cN0). SLNB is generally believed to yield the same prognostic outcome for patients as END. In this study, we corroborated the existing literature and demonstrated that SLNB is a safe alternative diagnostic tool that does not compromise outcomes [17,18,19,20,21,22].

In pN+ patients in the SLNB group, the overall survival, disease-free survival and disease-specific survival are significantly lower, despite subsequent END. We postulate that this could be significant, as patients undergoing END do not experience treatment delay in pN+ cases, whereas pN+ patients undergoing SLNB face a delay of several weeks before receiving END treatment.

Only a few studies exist regarding the impact of SLN metastases of OSCC on overall survival. In a meta-analysis, Ding et al. indicated that the prognosis for patients with SLNB is similar to that of patients with END. However, due to the significantly less invasive nature of SLNB, the associated risk is considerably lower [23]. In their investigations, Den Toom et al. showed that SLNB is equivalent to END, except in the case of tumors of the floor of the mouth [24]. The literature has consistently demonstrated that SLNB is as safe as END. However, the impact of the size of SLN metastases remains unclear. Broglie et al. concluded that while SLN metastases of any size impact survival, the size of the metastasis is correlated with survival [7]. The overall survival of patients with ITCs or micrometastases appears to be higher than that of patients with macrometastases, making the results of SLNB an adequate prognostic factor for survival [19]. Thomsen et al. reported that ITCs could include small micrometastases that are waiting to proliferate, suggesting that ITCs may hold significance in prognosis [25]. Because only one patient in our cohort had ITCs, it is not possible to draw any conclusions about their effects. However, this patient had local recurrence on the floor of the mouth. Questions surrounding the prognostic effects of ITCs and the best possible treatment, while relevant, remain unanswered. Hingsammer et al. suggested that ITCs may represent a risk factor for cervical lymph node metastases, but adequate proof of this is not available [17]. Other authors believe that the early detection of micrometastases or ITCs has a positive influence on the survival rate [26]. In contrast to these assumptions, a study by the University Hospital Montpellier showed that ITCs do not hold pathological impact [27].

An understandable fear of surgeons is missing metastases due to a false-negative SLNB result. In a large European study, a false-negative rate of 14% was observed [19].

Yao et al. presented an artificial intelligence (AI)-assisted prediction model, ACE-Net, which was able to effectively predict central lymph node metastasis in thyroid cancer. This tool is considered a valuable option for minimizing morbidity without missing positive cases and could support clinical decision-making regarding minimally invasive SLNB [28].

Our results demonstrate that patients treated with END following the diagnosis of early-stage SCC and cN0 exhibit excellent recurrence-free survival rates in both groups. Overall, there were no statistically significant differences in outcome between the groups. However, upon performing a subgroup analysis, the pN+ cohort demonstrated a higher incidence of recurrent disease.

Our study holds particular significance as it maintains consistency in the team of professionals treating the patients, ensuring a consistent level of experience throughout the study period. The observed variation in the percentage of recurrent disease is solely attributed to the change in the method used. While SLNB emerges as a valid option for stage 1 and 2 OSCC, the presence of occult metastases introduces a time delay before patients undergo then-necessary END, potentially contributing to poorer outcomes in these cases. pN+ patients who undergo delayed neck treatment subsequent to SLNB require special attention to mitigate any adverse effects.

Because END was more common before 2014 and SLNB predominated thereafter, in this study, our comparison is subject to potential temporal confounding. Advances in cross-sectional imaging, histopathological assessment and adjuvant therapy during the study period may have influenced detection of nodal disease and subsequent outcomes, introducing a source of bias that cannot be fully adjusted for in this retrospective design.

A key limitation of this study is the number of recurrence events (*n* = 15), which reduces precision of effect estimates. A post hoc power calculation indicated that, with a total of 15 events and an observed HR of 3.4, the achieved power to detect a difference in recurrence-free survival between groups was only ~36%.

The observation of a higher recurrence risk among pN+ patients treated with SLNB is clinically noteworthy but based on very few events and extremely wide confidence intervals. Accordingly, this finding requires confirmation in larger prospective, multi-institutional cohorts to support a fully adjusted survival analysis before influencing clinical decision-making.

SLNB holds potential value not only in early-stage OSCC but also in more advanced stages. While the university hospital of Zürich utilizes SLNB solely for nodal assessment in T1 and T2 tumors, research by Bark et al. suggests its applicability in T3 or T4 carcinoma cases as well [28]. Moreover, incorporating contralateral SLNB alongside ipsilateral END could enable the detection of metastases stemming from contralateral lymphatic drainage, thereby reducing the morbidity associated with bilateral neck dissection [29].

In conclusion, SLNB is a valuable tool for assessing ipsilateral lymph nodes in the neck, but continuous monitoring of disease-related outcomes is essential. Expanding the use of SLNB to various indications of head and neck cancer could enhance the detection of occult metastases in contralateral necks, further optimizing patient care.

## 5. Conclusions

Our study findings indicate that in early-stage OSCC (T1 or T2, cN0), SLNB is equally effective in terms of patient survival compared to END. We identified a possible increased risk of recurrent disease with delayed neck treatment in pN+ patients, but this needs to be confirmed in further clinical studies.

## Figures and Tables

**Table 1:** Distribution of the collected data.

		SLNB (*n* = 55)	END (*n* = 27)	Total (*n* = 82)
Age at surgery		57.2 (47.2–70.8)	59.9 (53.7–72.6)	58.9 (51.4–71.5)
Male		31 (56.4%)	15 (55.6%)	46 (56.1%)
Cancer location	Floor of the mouth	13 (23.6%)	18 (66.7%)	31 (37.8%)
	Tongue	42 (76.4%)	9 (33.3%)	51 (62.2%)
	Total	55 (100%)	27 (100%)	82 (100%)
Touching midline		13 (23.6%)	9 (33.3%)	22 (26.8%)
Laterality	Left	25 (45.5%)	12 (44.4%)	37 (45.1%)
	Center	4 (7.3%)	1 (3.7%)	5 (6.1%)
	Right	26 (47.3%)	14 (51.9%)	40 (48.8%)
pT classification	pT1	44 (80.0%)	17 (63.0%)	61 (74.4%)
	pT2	11 (20.0%)	10 (37.0%)	21 (25.6%)
pN classification	pN0	45 (81.8%)	20 (74.1%)	65 (79.3%)
	pN1	4 (7.3%)	3 (11.1%)	7 (8.5%)
	pN2a	1 (1.8%)	0	1 (1.2%)
	pN2b	3 (5.5%)	4 (14.8%)	7 (8.5%)
	pN2c	1 (1.8%)	0	1 (1.2%)
	NA	1 (1.8%)	0	1 (1.2%)
R0 resection		53 (96.4%)	27 (100%)	80 (97.6%)
Grade	G1	17 (30.9%)	1 (3.7%)	18 (22.0%)
	G2	30 (54.6%)	17 (63.0%)	47 (57.3%)
	G3	8 (14.5%)	9 (33.3%)	17 (20.7%)
Postoperative radiotherapy		3 (5.5%)	14 (51.9%)	17 (20.7%)
Total recurrence		13 (23.6%)	2 (7.4%)	15 (18.3%)
Recurrence in lymph nodes		3 (5.5%)	1 (3.7%)	4 (4.9%)
Time to recurrence (d)		1240 (476–1807)	1506 (1094–2035)	1326 (631–1847)

**Table 2:** Patients with recurrent disease and relevant characteristics.

Sex	Date of Surgery	Procedure (1 = Sentinel; 2 = ND)	pT	pN	Time to Recurrence (Months)	Location of Recurrence
m	20.02.2014	1	1	0	8.3	tongue
m	19.10.2009	1	1	0	66.4	floor of the mouth
m	25.11.2003	1	1	1	109	floor of the mouth
m	19.07.2012	2	1	0	47.1	floor of the mouth
m	19.05.2008	1	1	3	144.3	tongue
m	12.05.2009	1	1	0	138.9	tongue and floor of the mouth
m	17.12.2013	1	2	1	9	lymph node level II
m	06.11.2015	1	1	0	12.5	tongue
m	13.02.2018	1	2	0	1.5	lymph node levels I, II, III, IV, V
f	03.09.2018	1	1	3	15.7	soft tissue level VI/VII
m	21.12.2007	1	1	0	4.2	floor of the mouth
f	20.12.2016	2	2	0	15	lymph node level III
f	21.05.2019	1	1	0	18.0	anterior mandible
m	25.10.2019	1	1	0	15.1	lymph node level Ib

**Table 3:** Summary of recurrence patterns.

	SLNB	END
Total recurrence, n (%)	13 (23.6)	2 (7.4)
Site of first recurrence		
Local (tongue/floor of the mouth)	6	1
Regional/nodal (levels I-V/VI/VII)	5	1
Local + regional	1	0
Distant	1	0
Median time to recurrence (months)	23.6 (IQR 9–67)	31.1 (IQR 15–47)
Soft tissue level VI/VII		

**SLNB**

**END**

## Data Availability

The datasets generated and analyzed during the current study are not publicly available due to local regulations and the need to protect confidential patient information. However, deidentified data may be made available by the corresponding author upon reasonable request and subject to approval by the appropriate institutional and ethical review boards.
